# Severe Skin Forms of Psoriasis in Black Africans: Epidemiological, Clinical, and Histological Aspects Related to 56 Cases

**DOI:** 10.1155/2013/561032

**Published:** 2013-12-29

**Authors:** Komenan Kassi, Oussou Armel Mienwoley, Mohamed Kouyate, Sylvanus Koui, Kouame A. Kouassi

**Affiliations:** ^1^Department of Dermatology and Infectiology, Training and Research Unit of Medical Sciences, Félix Houphouët Boigny University (FHBU) of Abidjan-Cocody, Abidjan 21 BP 5151, Cote d'Ivoire; ^2^Training and Research Unit of Medical Sciences, University of Bouaké, Bouaké 01 BP V18, Cote d'Ivoire; ^3^Mother Maria Elisa Andreoli Health Center, Cocody Riviera Palmeraie, Cidex 3, Abijan-Riviera BP 51, Cote d'Ivoire; ^4^Department of Histopathology, University Hospital of Treichville, Abidjan 01 BP V3, Cote d'Ivoire

## Abstract

*Bacground*. Psoriasis is an erythematosquamous dermatosis of chronic development. In sub-Saharan Africa, few studies have been focused on complicated forms of psoriasis. *Objective*. The aim is to describe epidemiological, clinical, and histological features of severe skin forms of psoriasis in Cote d'Ivoire. *Material and Methods*. The study was both cross-sectional and descriptive, that focused on patient admitted to the dermatology unit for complicated psoriasis, from January 1st, 1986, to December 31th, 2007. *Results*. Fifty-six patients admitted to hospital for severe skin forms of psoriasis were recorded and included in our study over 7.503 patients hospitalized during the study period. They represented 0.75% of cases. The average age was 39.6 ± 3.3 years. There were 49 male (87.5%) and 7 female patients (12.5%) with a sex ratio of 7. At socioprofessional level, 48 patients (87.5%) were from category 1. Patients' history was dominated by the psoriasis vulgaris. Physical and general signs were dominated by itching (58.9%). The three severe skin forms were observed with predominant erythrodermic psoriasis (60.7%). Fifteen patients (34.9%) were HIV positive. *Conclusion*. Severe skin forms of psoriasis are rare in our setting. But in the quarter of HIV-positive patients, they are dominated by the erythrodermic psoriasis.

## 1. Introduction

Psoriasis is an erythematosquamous dermatosis of chronic development. It is ubiquitous and seems common in the West where prevalence rate ranges from 2 to 3% in the population at large [1, 2]. The benign forms are the most numerous, around 90%, and raise an aesthetic issue. The severe forms which are life-threatening or threaten the functional prognosis account for about 10% and require an admission to hospital. Psoriasis diagnosis is clinically easy in typical forms. Yet a histological confirmation is required after a biopsy of the skin lesion. Histological images are characteristic with parakeratotic hyperkeratosis and Munro-Sabouraud's microabscesses [2]. In sub-Saharan Africa few studies have been focused on complicated forms of psoriasis [3]. The objective of this study is to describe the epidemiological, clinical, and histological aspects of psoriasis complicated forms in patients admitted to the Dermatology Unit of Treichville University Hospital.

## 2. Material and Methods

The study was cross-sectional and descriptive. It focused on all the records of patients admitted to the Dermatology Unit of Treichville University Hospital for complicated forms of psoriasis in the period covering January 1, 1986, to December 31, 2007.

In the study records of patients of all sexes and ages suffering from erythrodermic psoriasis, universal psoriasis, or pustular psoriasis confirmed by histology were included. The histological examination has been carried out in the anatomopathology labs of Treichville University Hospital on a sample of the skin lesion biopsy. The sample was stored in 10% formalin. Paraffin inclusion was performed before examining under an optical microscope.

Epidemiological, clinical, and histological data along with HIV status have been recorded in a prescribed survey form.

The socioprofessional status has been subdivided into three categories.

- Category I: patients with monthly pay lower than the index-linked guaranteed minimum wage in Côte d'Ivoire (35,000 FCFA, i.e. 53,8 Euros).
- Category II: medium ranking civil servants of Ivorian public civil service with an average wage of 137 Euros [3].
- Category III: patients with more than 300 Euros as monthly wage.

Data analysis has been performed with the software Epi info 6.04 and has consisted in calculating the rates.

## 3. Results

We selected 56 records of patients suffering from complicated forms of psoriasis out of a total of 7503 hospitalization records during the 22-year study period representing 2.5 cases per year. Cumulative incidence of complicated forms of psoriasis was 0.7%. There were 49 male (87.5%) and 7 female patients (12.5%) which equals a sex ratio of 7. Average age was 39.6 ± 3.3 years with extremes of 4 and 77 years. There were 3 children (5.3%) and 53 adults (94.7%). From the adults, 38 patients (67%) were between 30 and 50 years old. At socioeconomic level, patients earning a monthly income less than 53.8 Euros accounted for 85.7% of cases. Our patients' history was dominated by psoriasis vulgaris in 21 cases (37.5%) followed by medication use in 18 cases (32.1%), tobacco in 14 cases (25%), alcohol in 14 cases (25%), combination of tobacco and alcohol in 8 cases (14.3%), stress in 3 cases (5.3%), and other variables in 7 cases (12.5%). Benzathine-penicillin and non steroidal topical remedies were the most used medecine in respectivily 22% of cases. Three severe forms have been observed. There were erythrodermic psoriasis in 34 cases (60.7%), universal psoriasis in 21 cases (37.5%), and generalized pustular psoriasis in 1 case (1.8%). Physical and general signs were dominated by itching in 33 cases (58.9%) (Table 2). Ungual impairments were observed in 33 patients (58.9%) and were dominated by “thimble-like” aspect in 13 cases (39.4%) followed bysubungual hyperkeratosis and pachyonychia in 4 cases (12.1%), onycholysis in 2 cases (6.1%), and others in 9 cases (27.3%). Patients were screened for HIV infection in 43 cases (76.8%). Fifteen patients (34.9%) were HIV positive with 9 cases of erythrodermic psoriasis and 6 cases of universal psoriasis. Histology involved 11 patients (19.6%). At the epidermic level, hyperkeratosis and agranulosis were observed in all the slides. Munro-Sabouraud abscesses were objectified in 8 slides (72.7%) (Table 1). Histological changes of the dermis were dominated by a lymphocytic infiltrate objectified in all the slides and a hyperpapillomatosis in 9 cases (81.8%) (Table 2).

## 4. Discussion

The severe psoriasis is rarely seen in the Dermatology Unit of Treichville University Hospital. This rareness has been reported by Kundakci et al. [4] in the Turkish in 2002 with an incidence of 0.07%. Our data is relatively low compared to those raised by Jalal et al. [5] in 2005 in Morocco and Lapeyre et al. [6] in 2007 in France with, respectively, 13 and 4.5 cases per year. This observation should confirm this disease rareness in blacks and particularly in West Africans [3, 7, 8]. Our study has pointed out a clear male predominance with 7 as sex ratio. This male predominance has been reported by many authors [5, 9, 10]. This marked difference observed in our study may be due to the delay in caring for the initially benign skin disorders in male patients for they show little concern for their physical look. Adults accounted for 94.7% and children 5.3%. They were adults of average age (39.6 ± 3.3 years old). This observation is in line with many authors' data [4, 5, 11]. Yet, Fortune et al. [12] in 2010 in Saudi Arabia have observed the severe psoriasis in adults of 22 to 26 years old. Kundakci et al. [4] reported the rareness of the disease in children (≤10 years old; 5.7%).

In our study, the severe psoriasis observed in adults may provide a proof of a late start of the (type II) disease in relation to non pustular clinical forms [1]. More than four-fifths of patients suffering from severe psoriasis were from category I. Public health facilities are visited by poor income patients in most cases due to the social rates charged whereas middle and high income patients would favor private health facilities. Moreover, we should not systematically reject the psychological impact of the bad living condition of category I patients on psoriasis beginning and/or worsening. According to Griffiths and Richards [11], psoriasis is a complex disease combining biological, psychological, and social contributors. Patients have different medical histories. Tobacco intoxication accounted for 25%. Alcohol consumption along with tobacco-alcohol combination accounted for 14.3% of cases. Dereure and Guilhou [8] mentioned tobacco and alcohol to be the psoriasis exogenous risks. Jalal et al. [5] found 8.9% of tobacco intoxication in patients suffering from severe psoriasis. In 2007, in Spain, Huerta et al. [13] reported that tobacco was an independent psoriasis risk factor. In 2008, Kirby et al. [10] observed link between psoriasis severity and weekly alcohol consumption. More than one-third of the patients suffering from severe psoriasis in our study have already been diagnosed with psoriasis vulgaris. The switch from psoriasis vulgaris to severe psoriasis is well known. Jalal et al. [5] in their series have reported a history of psoriasis vulgaris in 89.4% of cases. This precession of the severe psoriasis by the psoriasis vulgaris is caused by some contributors including drugs. Many authors have reported drug involvement in worsening or leading to psoriasis outbreak [2, 5, 6, 13]. Bérard et al. [2] have observed that mechanisms with which environment contributors such as drugs worsen or lead to outbreak are well known. Jalal et al. [5] reported an incidence of 12.5% of patients suffering from severe psoriasis triggered by the use of drug. Lapeyre et al. [6] observed that the erythrodermic psoriasis may be sparked by the introduction of new drugs. Huerta et al. [13] stated that antibiotics' use might cause an existing psoriasis to exacerbate.

The stress may be another contributor likely to lead to psoriasis outbreak or worsen an existing one according to some authors [2, 9, 11, 14]. In our study, 5.3% of patients have pointed out stress presence. However, Huerta et al. [13] stated that there was no connection between psoriasis occurrence risk and stress histories. The three severe skin forms of psoriasis have been observed in our study and are the erythrodermic psoriasis (60.7%), the universal psoriasis (37.5%), and pustular psoriasis (1.8%). Jalal et al. [5] and Lapeyre et al. [6] have reported the three severe skin forms of the psoriasis in their series at the following respective rates: erythrodermic psoriasis (54.4% and 14.2%), universal psoriasis (19.4% and 67.8%), and pustular psoriasis (19.4% and 18.0%). Kundakci et al. [4] reported the pustular psoriasis only with 17 cases. Fatani et al. [9] observed the erythrodermic psoriasis (57.9%) and the pustular psoriasis (42.1%). Our study pointed out rareness of the pustular psoriasis while this clinical form seems to be more common in the Maghreb, in Europe, and in the Middle East. This observation may suggest that there is a difference in the genetic factors associated with the onset of psoriasis between Caucasian and black African patients [8]. Severe skin forms of the psoriasis were accompanied with physical and general signs. Our study provided that there were itching (58.7%), oedema of lower limbs (28.6%), and hyperthermia (26.8%). Fatani et al. [9] have reported itching in 43% of cases. Globe et al. [15] have shown that itching was the most important sign and the most severe in the course of psoriasis. For patients included in this study, itching would cause an important deterioration of the quality of life. Jalal et al. [5] have found hyperthermia in 3% of patients. These physical and general signs are mostly observed in erythrodermic forms of psoriasis. The important impairment of the skin barrier may be the cause of biochemical disorders, thermoregulation changes, and hydroelectrolytic and protein disorders. More than half of our patients (58.9%) had ungual impairments dominated by “thimble-like aspect” (39.4%). Kundakci et al. [4] reported the ungual impairments in 16.4% of cases with “thimble-like aspect” dominating (79.6%). Mak et al. [14] asserted that almost half of the patients suffering from psoriasis had ungual impairment dominated by the “thimble-like aspect.” For some authors, HIV infection is a contributing factor to the occurrence of severe and extensive forms of psoriasis [8, 14, 16, 17]. According to most of these authors, there was no change in the incidence of psoriasis patients associated with HIV in comparison to the general population. But, in our series, 15 patients in 43 detected were HIV positive, that is, 34.9% of cases. The prevalence of patients suffering from severe psoriasis associated with HIV seemed high, in 28.8% of cases, yet the study does not allow checking whether severe psoriasis erupted before or after the HIV infection. This limit does not help to assess involvement of HIV infection in occurrence of severe forms of psoriasis. This prevalence observed in our study was lower than in the study conducted in eastern Africa where it was 41.6% of cases (in a population of 61 HIV-positive patients diagnosed for complicated psoriasis) [18]. However, it was higher than in Caucasians. In fact, a study in 2000 on HIV-positive patients showed a prevalence of 2.5% of cases compared to the general population in San Francisco [19]. Another study in Berlin on 700 patients infected by HIV reported a prevalence of 5% of cases, 3 times higher compared to the general population [16]. This high rate of HIV associated with severe forms of psoriasis in our setting could be explained by the high rate of HIV/AIDS incidence and prevalence in sub-Saharan Africa (the most infected region worldwide), in particular in Côte d'Ivoire.

## 5. Conclusion

Although they are scarce, severe forms of psoriasis are a concern to practitioners for being more often life-threatening because of the biological disorders and infectious complications they involve. Incidence of these severe cases of psoriasis in HIV-positive patients requires systematic HIV testing.

## Figures and Tables

**Table 1:** Histological epidermal changes in 11 severe psoriasis cases.

Epidermal changes	Number (*n*)	Percentage (*n*/11)
Hyperkeratosis	11	100.0
Parakeratosis	8	72.7
Parakeratosis and orthokeratosis	3	27.3
Microabscesses	8	72.7
Agranulosis	11	100.0
Thinning of the dermal papilla roof	10	90.9
Interpapillary bud changes		
Acanthosis	10	90.9
Elongation	8	72.7
Club aspect	7	63.6
Exocytosis	8	72.7
Polynuclear and mononuclear neutrophils	7	63.6
Mononuclear	1	9.1
Spongiosis	4	36.4
Minimal	3	27.3
Moderate	1	9.1

**Table 2:** Histological dermal changes in 11 severe psoriasis cases.

Dermal changes	Number (*n*)	Percentage (*n*/11)
Hyperpapillomatosis	9	81.8
Turgidity of dermal papilla	4	36.4
Presence of dilated capillaries	7	63.6
Superficial dermal infiltrate	11	100.0
Lymphocytes	11	100.0
Polynuclear	10	90.9
Neutrophil	8	72.7
Eosinophil	1	9.1
Neutrophil and eosinophil	1	9.1
Histiocytes	10	90.9
Plasmacytes	8	72.7
